# Sensitive Photodynamic Detection of Adult T-cell Leukemia/Lymphoma and Specific Leukemic Cell Death Induced by Photodynamic Therapy: Current Status in Hematopoietic Malignancies

**DOI:** 10.3390/cancers12020335

**Published:** 2020-02-02

**Authors:** Takashi Oka, Ken-ichi Matsuoka, Atae Utsunomiya

**Affiliations:** 1 Department of Hematology, Oncology & Respiratory Med., Graduate School of Medicine, Dentistry and Pharmaceutical Sciences, Okayama University, Okayama 700-8558, Japan; 2 Department of Hematology, Imamura General Hospital, Kagoshima 890-0064, Japan

**Keywords:** ATL, HTLV-1, PDT, PDD, chemotherapy, allogeneic hematopoietic cell transplantation, immunotherapy, GVHD, ALA-PDT/PDD

## Abstract

Adult T-cell leukemia/lymphoma (ATL), an aggressive type of T-cell malignancy, is caused by the human T-cell leukemia virus type I (HTLV-1) infections. The outcomes, following therapeutic interventions for ATL, have not been satisfactory. Photodynamic therapy (PDT) exerts selective cytotoxic activity against malignant cells, as it is considered a minimally invasive therapeutic procedure. In PDT, photosensitizing agent administration is followed by irradiation at an absorbance wavelength of the sensitizer in the presence of oxygen, with ultimate direct tumor cell death, microvasculature injury, and induced local inflammatory reaction. This review provides an overview of the present status and state-of-the-art ATL treatments. It also focuses on the photodynamic detection (PDD) of hematopoietic malignancies and the recent progress of 5-Aminolevulinic acid (ALA)-PDT/PDD, which can efficiently induce ATL leukemic cell-specific death with minor influence on normal lymphocytes. Further consideration of the ALA-PDT/PDD system along with the circulatory system regarding the clinical application in ATL and others will be discussed. ALA-PDT/PDD can be promising as a novel treatment modality that overcomes unmet medical needs with the optimization of PDT parameters to increase the effectiveness of the tumor-killing activity and enhance the innate and adaptive anti-tumor immune responses by the optimized immunogenic cell death.

## 1. Introduction

Light treatments have been used as therapy for over 3000 years [1,2]. Specifically, light treatment has been in use for treating various diseases (including psoriasis, rickets, vitiligo, and skin cancer) in ancient Egyptian, Indian, and Chinese civilizations [3]. Hematoporphyrin (HP), which is a crude porphyrin extract from blood has fluorescence property, and the fluorescence was used for tumor detection since about 100 years ago [4,5]. The photosensitizing property of porphyrins, which absorb light energy and converts it into the production of cytotoxic singlet oxygen (^1^O_2_) and other reactive oxygen species (ROS) in the presence of oxygen, was recognized in the 1900s and extensively studied using partially purified HP, since the 1970s [3,4,5,6]. Photodynamic therapy (PDT) is based on the principle of a selective uptake of a nontoxic drug or dye: called the photosensitizer, which specifically localizes to tumor cells and/or malignant tissues, then the lesion is illuminated with low-energy tissue-penetrating light that is of appropriate and specific wavelength, usually visible light, in the presence of oxygen. Eventually, tumor cell death and tissue disruption results, following the generation of singlet oxygen and other reactive oxygen species (ROS). PDT has been used since the early twentieth century, employing dyes such as eosin together with light to treat cutaneous disorders especially skin cancer; however, the range of indications has recently significantly expanded, including the brain, bladder, endobronchial, esophageal and gastric cancers [3,4,5,6,7,8,9,10,11].

Intrinsic biochemical and metabolic molecules in the body that are localized within tumor tissues have been used as light-activated therapeutic targets. The first metabolite in the heme biosynthesis pathway in humans is 5-Aminolevulinic acid (5ALA). This pathway also includes several porphyrin metabolites in addition to the end product, heme. Protoporphyrin IX (PpIX) is an immediate heme precursor porphyrin that has efficient fluorescence and photosensitizing activity. PpIX absorbs energy directly from an innocuous visible light source as a natural photosensitizer, and then transfers the energy to molecular oxygen to create ^1^O_2_ and other ROS. The ^1^O_2_ is one of the cytotoxic molecules that reacts promptly with cellular constituents, thus inducing tumor cell impairment that eventually leads to tumor cell death with necrosis and/or apoptosis and tumor disruption [5,6,7]. 5ALA has been studied for the detection and treatment of tumors in some organs. Application of 5ALA as a diagnostic tool is based on the selective accumulation of the heme precursor PpIX in tumors and precancerous lesions. Photodynamic diagnosis (PDD) and its clinical applications were intended to provide a good definition of surgical margins in brain or skin tumors to better detect flat precancerous lesions as well as for the early detection of tumors in the breast, bladder, endobronchial tissues, and gastrointestinal tract [8,9,10,11,12]. 

An ideal cancer treatment approach comprises not only of the primary tumor killing, but also of initiating the tumor immune response to simultaneously recognize, pursue, and kill any residual malignant tumor cells at or near the site of the primary tumors, as well as any distant metastases. Practically, almost all the commonly used cancer treatments are immunosuppressive. At sufficient doses to destroy the tumors, chemotherapy, and X-irradiation treatments are also toxic to the bone marrow, the source of all the cells of the immune system. At a certain level, the dose-limiting toxic side effects of these treatments, including neutropenia and other forms of myelosuppression, become evident. There are several potential advantages of PDT over radiotherapy, chemotherapy, and surgery. PDT has a relatively non-invasive property, can be targeted precisely and repeated doses are applicable without the limitations of total doses that accompany radiotherapy and chemotherapy. Also, this can be implemented with little or no scarring following a moderate healing process. Moreover, day-case settings or outpatients are usually feasible with PDT treatments. That is, it is convenient for the patients, and has no serious side effects [3,6,7,13,14,15].

For more than 35 years, the clinical potential of ALA-PDT has been recognized. However, ALA-PDT applications are still at the initial stages [6,13,14,15]. This is mainly because the light is not able to penetrate into the tumor tissues beyond the 3 mm thickness to induce sufficient tumor necrosis and/or apoptosis. Additionally, a longer incubation time is required for ALA-PDT between drug applications and light exposures, in order to be metabolically converted into PpIX. Moreover, investigations into PDD and PDT using clinical specimens for hematopoietic malignancies such as leukemia and lymphoma have remained at its early stages, so far.

This review presents an overview of the current status of PDD and PDT on hematopoietic malignancies including leukemia and lymphoma, particularly focusing on adult T-cell leukemia/lymphoma (ATL). 

## 2. ATL: Current Treatment and Remaining Issues

### 2.1. Current Treatment

**Figure 1:**
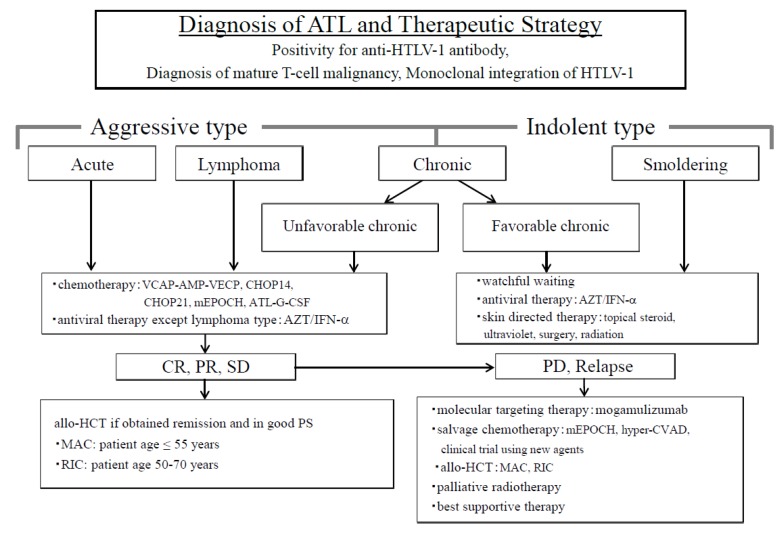
Diagnosis of adult T-cell leukemia/lymphoma (ATL) and the therapeutic strategy. ATL is divided into two types in order to decide treatment strategy; one is an aggressive type and the other is an indolent type. The aggressive types are acute, lymphoma, and unfavorable chronic, while the indolent types include the favorable chronic and smoldering types. Therapeutic strategies are decided based on these classifications. Allo-HCT, allogeneic hematopoietic cell transplantation; ATL-G-CSF, combination chemotherapy consisting of vincristine, vindesine, doxorubicin, mitoxantrone, cyclophosphamide, etoposide, ranimustine, and prednisone with granulocyte-colony stimulating factor support; AZT⁄IFN-α, zidovudine and interferon-α; CHOP, cyclophosphamide, doxorubicin, vincristine, and prednisone (CHOP14 is performed every 2 weeks, and CHOP21 is performed every 3 weeks); CR, complete remission; hyper-CVAD, cyclophosphamide, vincristine, doxorubicin, and dexamethasone; MAC, myeloablative conditioning; mEPOCH, etoposide, prednisone, vincristine, cyclophosphamide, and doxorubicin (EPOCH) with modifications; PD, progressive disease; PR, partial remission; PS, performance status; RIC, reduced-intensity conditioning; SD, stable disease; VCAP−AMP−VECP, vincristine, cyclophosphamide, doxorubicin and prednisone (VCAP)-doxorubicin, ranimustine and prednisone (AMP)−vindesine, etoposide, carboplatin, and prednisone (VECP). Figure 1 was reproduced and modified from Figure 1 in Utsunomiya et al. (*Cancer Science*, 2015) [19]. Reprint is permitted by *Cancer Science*.

ATL is an intractable peripheral T-cell malignancy caused by the human T-cell leukemia virus type-I (HTLV-1) which is characterized by multiple organ invasions by ATL cells, a high frequency of opportunistic infections, the resistance of chemotherapeutic drugs, and poor prognosis [16,17]. ATL is classified into two types based on the treatment strategies to be implemented; one is the aggressive type, which includes the acute, lymphoma and unfavorable chronic types, and the other is the indolent type, which includes favorable chronic and smoldering types [16,18]. Patients with aggressive- type ATL require urgent treatment because of systemic symptoms and rapid progression. We have proposed a therapeutic strategy for patients with ATL (Figure 1) [19].

Complete remission (CR) rates for patients with aggressive ATL receiving combination chemotherapy is very low, ranging from 17% to 43%; the median survival time (MST) is also very short, ranging from 5 to 13 months, as reported by the Lymphoma Study Group (LSG) of the Japan Clinical Oncology Group (JCOG) [17,19,20,21,22,23]. The MST and 5-year overall survival (OS) rate of all patients who had registered in these 3 clinical trials (JCOG9109, JCOG9303, JCOG9801) were only 11 months and 14%, respectively [24].

Combination therapy is frequently used for patients with ATL receiving zidovudine and interferon-α (AZT/IFN-α) antiviral therapy in countries other than Japan. The high efficacy of this combination therapy for patients with indolent-type ATL has been reported by Bazarbachi et al. [25], and they also reported that the OS rate in patients with acute-type ATL who received this combination therapy was better than that in patients who received chemotherapy [25]. However, it was observed that the OS rate in patients with acute-type ATL who received AZT/IFN-α was not as favorable as that in Japanese patients with ATL who had been treated with intensive chemotherapy in the trials conducted by JCOG-LSG [21,23,24,25,26]. In Japan, a randomized phase III study comparing AZT/IFN-α to watchful waiting is currently ongoing only for patients with indolent-type ATL. Combination therapy of arsenic trioxide and AZT/IFN-α was reported to be effective in patients with newly diagnosed chronic-type ATL by Kchour et al. [27].

Dose-intensified chemotherapy using autologous stem cell support for patients with aggressive ATL failed to improve their outcomes because of frequent ATL relapses and a high incidence of infectious complications [28]. We first reported the possibility that allogeneic (allo) hematopoietic cell transplantation (HCT) using myeloablative conditioning (MAC) may improve the outcome of patients with aggressive ATL [29]. Afterward, many methods of allo-HCT for patients with ATL using reduced-intensity conditioning, peripheral blood stem cells, and cord blood were developed by Japanese researchers [30,31,32,33]. Furthermore, HLA-haploidentical HCT was performed in patients with ATL who did not have HLA-matched suitable donors [34]. HLA-haploidentical HCT using post-cyclophosphamide administration is currently under investigation in patients with ATL in Japan. Many nationwide retrospective analyses of allo-HCT in patients with ATL were performed using the Japanese Transplant Registry Unified Management Program database [35]. Although OS rates of patients with ATL are not so high, allo-HCT is regarded as the only curative treatment for aggressive ATL in Japan at the present time. Furthermore, allo-HCT is thought to be the curative treatment in many countries other than Japan [33,36,37]. Recently, two new molecular targeting agents have been approved for aggressive ATL in Japan. One is anti-CC chemokine receptor 4 (CCR4) antibody, mogamulizumab, which is effective in CCR4-positive patients with aggressive ATL, and the other is lenalidomide, which shows high response rates in relapsed patients with aggressive ATL [38,39,40].

Mogamulizumab in combination with chemotherapy for patients with newly diagnosed aggressive ATL showed a higher CR rate than that in patients receiving chemotherapy alone [41]. However, the use of mogamulizumab before allo-HCT showed a negative impact on OS in patients with ATL who underwent allo-HCT because of a high incidence of severe acute graft-versus-host disease (GVHD) and high transplant-related mortality [42]. Mogamulizumab has a strong cytotoxic effect that can kill not only ATL cells, but also CCR4-positive normal regulatory T (T-reg) cells; therefore, severe acute GVHD might be induced in association with reduced T-reg cells in recipients [43]. An appropriate use of mogamulizumab for patients with ATL who plan to undergo allo-HCT is now developing in Japan [44]. In patients with ATL who have CCR4 mutations of ATL cells, mogamulizumab therapy confers better OS compared to that in patients without CCR4 mutations [45].

Lenalidomide is another new drug that has been approved for relapsed patients with ATL in Japan [40]. The efficacy of lenalidomide for patients with ATL is expected to be similar to that of salvage therapy. However, the usefulness of lenalidomide during, before and/or after allo-HCT in patients with ATL has not been elucidated. This issue should be addressed by future research. 

### 2.2. Remaining Issues

We recognize the fact that a Tax-specific T-lymphocyte (CTL) response is associated with graft-versus-ATL effects in patients with the acute type of ATL after allo-HCT [46]. Vaccine therapy with Tax-peptide-pulsed dendric cells is expected to maintain a long-lasting remission in patients with aggressive ATL. A phase Ia/Ib study of this vaccine with Tax-targeted dendritic cells showed good efficacy in patients with aggressive ATL that stabilized after chemotherapy [47,48]. A phase II study of this vaccine therapy will commence in Japan soon. As for the other immunotherapy, immune checkpoint inhibitors such as programmed cell death-1 (PD-1) inhibitors and programmed cell death-ligand 1 (PD-L1) inhibitors are also expected to show high efficacy for patients with ATL. However, it has been reported that patients with indolent ATL progress rapidly after receiving PD-1 inhibitor (nivolumab) therapy [49,50]. In a small Japanese study of patients with aggressive ATL, no patient showed characteristics similar to those in the cases reported by Ratner et al. [49,50,51]. An anti-tumor role of the PD-1/PD-L1 pathway might be different between the indolent and aggressive stages of ATL. However, this trial of an anti-PD-1 antibody (nivolumab) should be given special attention. 

Recently, the genomic landscape of ATL has been clarified by Kataoka et al. based on an integrated molecular analysis [52]. They have also reported the relationship between genetic alterations and the prognosis of patients with ATL [53]. These analyses will contribute to the accurate diagnosis and the establishment of individualized therapeutic strategies in patients with ATL.

## 3. Basic Understandings of Photodynamic Therapy for ATL

### 3.1. Photodynamic Detection (PDD) for ATL Cells

Hematopoietic cell lines have been used for the investigation of the biological effects of PDT [54,55,56,57,58]. However, studies on PDD have been concentrated on solid tumors [1,2,3]. To our knowledge, the first study of PDD for human hematopoietic malignancies has been reported using human ATL patient specimen [59].

ATL, an aggressive malignant disease of the CD4 (+) T lymphocytes is induced by the HTLV-1 infection [60,61,62,63]. Globally, it is estimated that there are about 20 million HTLV-1-infected persons [64], and 1.1 million live in Japan. Approximately 1000 cases of ATL are estimated as the annual number in Japan alone [65]. HTLV-1 infections mainly occur via breastfeeding, resulting in ATL in about 3–5% of HTLV-1 asymptomatic carriers (ACs) after a prolonged latent period of 40–60 years. Such a long latent period suggests the possibility of a multi-step leukemogenic and/or lymphomagenic mechanism in the development of ATL [66]. About 95% of ACs preserve the AC state with no ATL developing throughout their lives. However, carriers have to live with the fear of possible ATL development. Therefore, one of the key issues revolves around the need to establish an accurate and sensitive method to identify high-risk ACs who are highly susceptible to developing ATL, so that they can be examined intensively and provided with preventive treatments for ATL. However, some indolent ATL patients may develop an acute crisis, which suggests the progression to the aggressive ATL. Almost 50% of aggressive ATL patients die within 6 to 12 months despite intensive intervention [19,67]. Consequently, the challenge that needs to be addressed urgently is for an established method to identify high-risk indolent ATL patients, who might have developed and/or are likely to develop the aggressive ATL, and also to establish practical clinical treatments to avoid an acute crisis.

**Figure 2:**
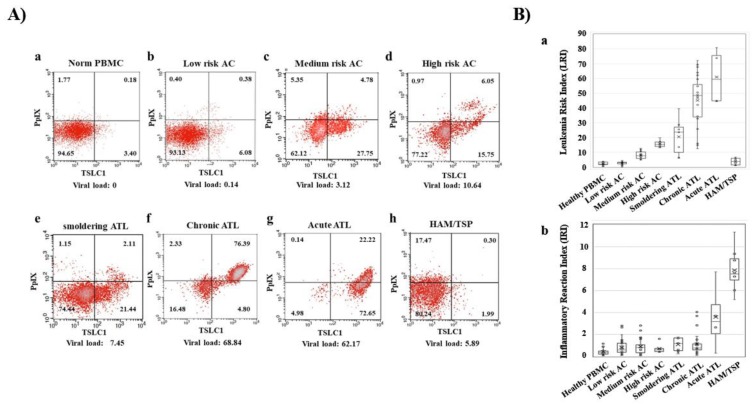
Dynamic changes of Flow cytometry (FCM) profiles during onset and progression of ATL. (**A**) FCM profiles with the Protoporphyrin IX (PpIX)/TSLC1 parameters indicating the dynamic changes during the onset and progression of ATL. (b–d) asymptomatic carrier (AC) peripheral blood mononuclear cells (PBMCs) profiles showed three patterns: Low-risk ACs (similar to healthy profile), medium-risk ACs (intermediate profile), and high-risk ACs (similar to smoldering ATL profile). (**B**) Leukemia Risk Index (LRI) and Inflammatory Reaction Index (IRI) changes in healthy donors, ACs and three types of ATL and HTLV-1-associated myelopathy/tropical spastic paraparesis (HAM/TSP). (a) ATL showed onset and progression-dependent increase in LRI. AC PBMCs were classified into three categories (low-, medium-, and high-risk) according to the LRI values, corresponding to the typical FCM profiles in A). (b) HAM/TSP showed high IRI values. This figure was reproduced and modified from Figure 3 in Oka et al. (*Scientific Reports* 2018) [59]. Reprint is permitted by *Scientific Reports*.

In recent times, preferential accumulation of the endogenous photosensitive metabolite, PpIX, has been shown in ATL cell lines, after a short-term culture with 5ALA. More than 10- to 100-fold accumulation of PpIX was observed in ATL leukemic cell lines and ATL patients’ leukemic cells compared to that in healthy peripheral blood mononuclear cells (PBMCs) after a short-term culture with 5ALA [59]. Dynamic changes in flow cytometry (FCM) profiles during the onset and progression of ATL were detected. PBMCs from healthy volunteers; ACs; and patients with smoldering-, chronic-, acute-type ATL, and HAM/TSP (HTLV-1-associated myelopathy/tropical spastic paraparesis) were analyzed with flow cytometry, using PpIX and TSLC1/CADM1 (tumor suppressor in lung cancer 1/cell adhesion molecule 1) parameters. Dynamic changes in 2D FCM profiles were shown from smoldering-, to chronic-, to acute-type ATLs in accordance with progression (Figure 2A). Leukemia Risk Index (LRI) was defined in order to clarify the profile differences in FCM data. According to the LRI data, asymptomatic HTLV-1 carrier PBMCs were classified into three categories: Low-risk ACs (similar to healthy profile), medium-risk ACs (intermediate profile), and high-risk ACs (similar to smoldering ATL profile). While the increase in LRI was observed to be progression-dependent from HTLV-1 carriers to indolent ATL, and acute ATL states, HAM/TSP, which is a chronic inflammatory disease, demonstrated that the LRI is almost similar to that of the low- or medium-risk ACs (Figure 2B). Furthermore, metabolomics analyses of the porphyrin pathway showed the preferential accumulation of the endogenous photosensitive metabolite, PpIX in ATL. Significant changes in the intermediate metabolites such as 5ALA, porphobilinogen, uroporphyrinogen III and coproporphyrinogen III were not detected in the normal CD4 (+) T cells, HTLV-1 immortalized cells, and ATL leukemic cells in cultures with and without 1 mM 5ALA. On the other hand, dramatic differences in PpIX levels were detected among them. More than several hundred-fold higher PpIX levels were found in the ATL leukemic cells and PpIX were 10–30-fold higher in ACs cells than that in the normal CD4 (+) T cells when cultured in the presence of 1 mM 5ALA. No accumulation of PpIX was detected in any of these cells in the control culture conditions without 5ALA. Additionally, downstream heme molecules; dihydrobiliverdin, and bilirubin, showed similar profiles with PpIX. This evidence indicated that the specific stage from PpIX to heme in the heme-metabolic pathway progressively deteriorated with HTLV-1 infection and at the onset and progression of ATL [59].

Then, PDD of PpIX in combination with the ATL leukemic cell marker would be useful to detect the minor population of leukemic cells in ACs and/or indolent ATL patients as well as for detection of overt stage acute ATL, which contributes to early detection of high-risk ACs and high-risk indolent ATL. PDD of PpIX could supply the valuable information on patient status and monitoring the dynamic changing behavior of leukemic cells or minimum residual disease after treatments in the single cell resolution. As the significant increase of PpIX have been detected in various kinds of leukemia and lymphoma cells after a short-term culture with 5ALA, including T-cell, B-cell leukemia/lymphomas, myeloid leukemias and also lymphomas, PDD of PpIX in combination with malignancy/disease-specific markers would be useful for the precise diagnosis of various hematopoietic malignancies and also inflammatory diseases.

### 3.2. Photodynamic Therapy (PDT) of ATL Cells

Generally, the published reports of PDT utility for leukemia have almost exclusively been performed with cultured cell lines [68,69,70,71,72]. To our knowledge from a database search, there are limited published studies on PDT in vivo or ex-vivo experiments for the treatment of human leukemia. One study assessed the treatment with extracorporeal photochemotherapy (photopheresis) in four patients with chronic or smoldering type ATL, which reported that the skin lesions with ATL cell infiltration began to disappear from 4 to 8 months after starting photopheresis. Cell surface markers in three of the patients exhibited improvement, while, in the remaining patient, serial decreases in soluble interleukin-2 receptor levels (950 U/mL to 620) occurred in the serum after 4 months. Based on the findings of this pilot investigation, application of photopheresis may be successful in ATL [73]. 

**Figure 3:**
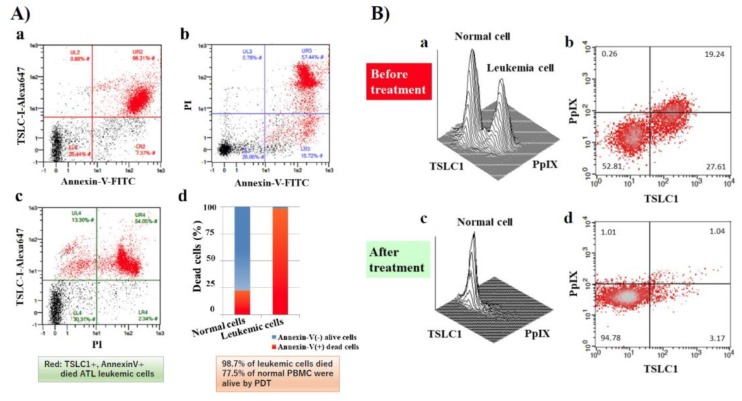
FCM analyses of chronic ATL patient PBMCs before and after photodynamic therapy (PDT). After PDT treatment, cells were labeled with PI, tumor suppressor in lung cancer 1 (TSLC1)-Alexa647, and Annexin V-FITC and analyzed. (**A**) (d) FCM analyses showed that 98.7% of TSLC1(+) ATL leukemic cells were TSLC1(+)/Annexin V(+) dead cells (red), whereas 77.5% of TSLC1(-) normal cells were TSLC1(-)/Annexin V(-) live cells (blue) after aminolevulinic acid (ALA)-PDT treatment, indicating that ALA-PDT induced highly-specific ATL leukemia cell death with minimal damage to normal PBMCs. FCM analyses of chronic ATL patient specimens before and after ALA-PDT treatment. (**B**) (a,b) Chronic ATL PBMCs incubated in 1 mM 5ALA for 48 h showing 2 peaks corresponding to the normal and ATL leukemic cells in TSLC1-FITC and PpIX profiles. (c,d) After 10 min of light exposure-treatment, the ATL leukemic cell peak completely disappeared and only the normal cell peak remained. This figure is reproduced, modified from Figures 5 and 6 in Oka et al. (*Scientific Reports* 2018) [59] Reprint is permitted by *Scientific Reports*.

In our recent publication, we showed that ALA-PDT successfully induced ATL leukemic cell death. ATL leukemic cell lines cultured in the presence of 1 mM 5ALA treated with 10 min of visible light exposure followed by FCM analyses using PI and Annexin V-FITC staining, showed that almost all ATL leukemic cells died by apoptotic and/or necrotic cell death after PDT treatment. Furthermore, 98.7% of ATL leukemic cell death could be induced with PBMC specimens from chronic ATL patients subjected to the same treatment, while 77.5% of normal PBMCs survived (Figure 3A(a–d)). Two clear peaks, corresponding to normal and ATL leukemic cell populations, respectively, were observed without light exposure conditions in the PpIX/TSLC1 profile of FCM (Figure 3B(a,b)). On the contrary, ATL leukemic cell peak completely disappeared and only a single peak corresponding to the normal cells remained after the light exposure, showing almost the same PpIX/TSLC1 profile of FCM as that of low- or medium-risk ACs (Figure 2; Figure 3B(c,d)). Essentially the same results as that of the PBMC experiments were reproduced with whole blood PDT experiments using chronic ATL patient blood specimens. This is because chronic ATL is an indolent type of ATL that may progress to an aggressive type of acute ATL. This evidence clearly shows that ALA-PDT treatment successfully eliminated ATL leukemic cells with highly-specific leukemia cell death via apoptosis and/or necrosis with minimal damage to normal PBMCs even in whole blood specimens, indicating the possibility ALA-PDT/PDD can inhibit the progression of ATL from indolent to aggressive types. Moreover, PpIX (+)/TSLC1 (+) ATL leukemic cells and intermediate pre-leukemic cells were sensitively detected by ALA-PDD for precise diagnosis, suggesting the possible role of ALA-PDT/PDD in preemptive therapy of ATL by eliminating the small population of leukemic and/or pre-leukemic cells in high-risk indolent ATL before the overt onset of aggressive ATL. Additionally, elimination of leukemic and/or pre-leukemic ATL cell population from high-risk ACs with ALA-PDT/PDD would be hopeful. The ALA-PDT/PDD along with the circulatory system may help with the diagnosis and treatment of various types of malignancies other than ATL, such as other types of lymphoid/myeloid leukemia. 

PDT kills malignant tumor cells by apoptosis and/or necrosis, and also induces various effects in the tumor microenvironments. These effects on the tumor-associated or -infiltrating immune cells take the lead in infiltrating various kinds of immune cells, for instance, the monocytes/macrophages and neutrophils, into the targeted sites. Immunogenic cell death also stimulates the host immune system, causing acute inflammation to release various kinds of acute-phase response and proinflammatory mediators, such as chemokines, HSPs, complement proteins, arachidonic acid derivatives, and cytokines (e.g., IL-1, IL-6, and TNF-α) [7,74,75]. Danger signals, called damage-associated molecular patterns (DAMPs), are produced from PDT-treated dying cells. DAMPs enhance antigen presentation by dendritic cells (DCs) and the recruitment of antigen-specific CD8(+) CTLs [7,74,75,76,77]. LCL521, acid ceramidase inhibitor, enhanced PDT, and PDT-generated vaccine effects have an effective restriction of the myeloid-derived suppressor cells, (MDSCs), and Tregs activities [78,79]. Antibodies against PD-1 and PD-L1, the immune checkpoint proteins, are a novel modality of therapeutic drugs for the treatment of cancers. The combination of ZnP@pyro PDT treatment with anti-PD-L1 consequently induces the eradication of light-irradiated primary tumors and furthermore the complete inhibition of untreated distant tumors by enhancing the systemic tumor-specific cytotoxic T cell response [75,80]. Further research will be able to optimize various PDT-related parameters.

## 4. Bench to Bed; Clinical Applications of PDT for ATL and Others

### 4.1. PDT for ATL Cells

Based on the findings described above, we are now at the stage of preparing clinical applications for this treatment. Although clinical treatments for aggressive ATL have been expanded over these years, they are still insufficient. Particularly, there are two main problems remaining in the current treatment of ATL; the first is the acquisition of resistance to conventional therapy during induction therapy and the second is the lack of treatment options at the time of recurrence. PDT is expected to solve these problems since it has the efficient and distinct cytotoxic mechanism that is clearly different from that of conventional treatments. As a bridge to allogeneic HCT, patients need to receive intensive combination chemotherapy to reduce the tumor burden; however, many cases could become refractory to chemotherapy before transplant. Although the efficacy of anti-CCR4 antibodies and immunomodulatory drugs such as lenalidomide have been approved for aggressive ATL, the pretransplant use of these drugs could cause severe GVHD after HCT, and thus, it is not appropriate as a bridging therapy to HCT [42,81]. As compared to anti-CCR4 antibodies or lenalidomide, the effect of PDT on normal immune cells appears to be negligible, the adverse effect of pretransplant PDT on GVHD after transplant is considered to be limited. Combining PDT with the conventional induction chemotherapy may enable faster and deeper remissions, which leads to safe transplant. On the other hand, it is also important to develop alternative treatments for refractory or recurrent diseases. We confirmed that in vitro experimental ALA-PDT could exert cytotoxic activity on ATL cells freshly obtained from patients with aggressive ATL which is clinically resistant to conventional chemotherapy or anti-CCR4 antibodies [82], suggesting that ALA-PDT can be a treatment modality for refractory or recurrent ATL patients after receiving the existing conventional treatments.

**Figure 4:**
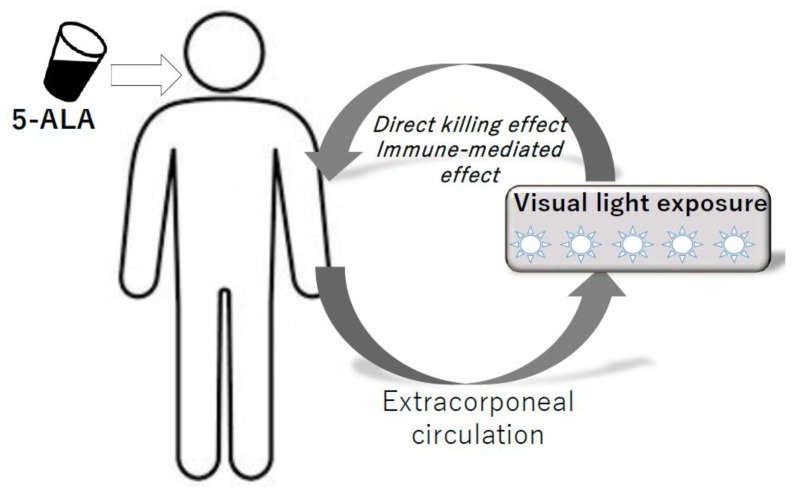
Model of ALA-PDT for hematopoietic malignancies using extracorporeal circulation system.

For the actual clinical applications, it is necessary to devise an in vivo system of PDT that can be used to expose circulating hematological cancer cells to the light. Tumors that have been targeted by PDT so far have been solid cancers such as skin cancer, esophageal cancer, and bladder cancer that can be easily exposed to light [83,84,85]. In contrast to solid tumors, the application of PDT to hematological malignancies is a challenging process because basically these do not present on the skin or luminal surface where light can reach. In this regard, previous studies on animal experiments about in vivo PDT system for hematological malignancies [57,86,87,88] and clinical studies of extracorporeal phototherapy using ultraviolet (UV-ECP) for mycosis fungoides (MF) and Sezary syndrome (SS) provide promising suggestions [89,90]. One preclinical study of an in-vivo animal model of PDT demonstrated that hematological cancer cells in peripheral blood could be directly killed by PDT. These studies indicate that direct killing can be observed in in-vivo environments where the light-shielding effect by erythrocytes may be interrupted with the cytotoxic effect of PDT on tumor cells. However, the direct killing alone does not provide sufficient clinical effect on blood cancers that have tumors in sites other than peripheral blood, and thus, a so-called abscopal effect is necessary. To consider this, the clinical observations that PDT using the extracorporeal circulation system has been effective for MF/SS may be giving us hints. UV-ECP is a form of phototherapy where blood is exposed extracorporeally to the photoactivated drug 8-methoxypsoralen (8-MOP). MF/SS was originally a dermatological hematologic tumor that was effective with direct skin irradiation, but PDT using an extracorporeal circulation system was performed on patients with a stage after systemic invasiveness and it has been reported that it works well beyond the direct killing effects. Even when just a limited portion of the total blood cells is treated by one course of the UV-ECP procedure, the usual experience is that of a larger reduction in malignant T cells [91]. Such a finding suggests that ECP may induce an immune-mediated response to malignant cells, as well as direct killing. The possible mechanism is proposed as follows: the induction of apoptosis of malignant T cells, maturation of dendritic cells (DCs), presentation of tumor-loaded DCs to CTL, and expansion of a population of CTLs against the malignant cells [89,92,93,94]. Similar biological effects have also been reported in ALA-PDT [95,96,97]. ATL is known to be sensitive to immunotherapy and may be a good target for PDT as an immune modulation therapy. We are currently planning a Phase1 clinical trial of ALA-PDT using extracorporeal circulation for aggressive ATL (Figure 4).

5ALA is administered orally, and then visible light is exposed to ATL cells and normal lymphocytes in peripheral blood using the extracorporeal circulation system. Direct killing effects and immune-modulating effects are expected.

There are several remaining issues to overcome for the start of clinical trials. First, to safely perform extracorporeal circulation, careful consideration of patient eligibility is required since patients suffering from aggressive ATL are often in poor general condition. Second, in the development of an extracorporeal circulation device, it is necessary to estimate the light shielding effect of red blood cells in peripheral blood and determine an appropriate amount of light irradiation. Third, the influence of PDT on normal hematopoietic cells in human clinical situations should be accurately evaluated. Because hematological cancer cells flow in the circulating bloodstream together with normal blood cells, it is impossible to specifically irradiate tumor cells with PDT. Each normal lymphocyte subset has its own specific function and the unpredicted depletion or modulation of the function of a certain subset can disrupt the overall immune balance.

In summary, although various problems remain, ALA-PDT can be promising as a novel treatment modality that overcomes unmet medical needs in current ATL treatment by having a direct-killing effect with distinct cytotoxic mechanisms and a possible abscopal antitumor effect by harnessing host immunity.

### 4.2. Other Possible Clinical Applications of ALA-PDT

UV-ECP has been used for cutaneous T cell lymphoma (CTCL) [98]. As in most reports on UV-ECP for CTCL, its use was in combination with other agents and modalities (including retinoids, PUVA, interferons, and others); indicating that ECP can be safely combined with many other therapeutic agents. ALA-PDT may have a possibility to apply CTCL as well. Also, ALA-PDT may be used for the prevention of metastasis of solid cancers because it could be a unique approach to eliminate circulating tumor cells (CTCs) from the bloodstream [99].

Other indications where ALA-PDT can be used are GVHD and solid organ transplant rejection. Chronic GVHD is an immune complication that occurs in 30–50% of patients who have undergone allogeneic HCT. Although it crucially affects the mortality and morbidity of long-term survivors after transplant, there is no established secondary treatment for steroid-refractory cases. Various treatments such as UV-ECP, iburutinib, ruxolitinib, and low-dose IL-2 have been studied for chronic GVHD [100,101]. Previous studies reported that UV-ECP can induce tolerance by modulating immune cells [102]. We studied the effect of ALA-PDT on allo-activated T cells using murine allogeneic BMT model and found that the activated donor-derived effector T cells acquired PpIX after exposure of 5ALA and was susceptible to apoptosis by PDT, suggesting ALA-PDT can reduce GVHD-responsible effector T cells and might improve clinical symptoms of GVHD [82]. ALA-PDT can be used with other immune-suppressive therapies and may offer a promising basic modality for patients with refractory chronic GVHD in the future.

## 5. Conclusions and Perspectives

The treatment of leukemia with PDT is an interesting approach because the side effects of PDT are modest in comparison to current chemotherapy and radiotherapy for leukemia. PDT is a relatively non-invasive approach. Therefore, it can be targeted precisely, and with repeated treatments, the limitations of chemotherapy and radiotherapy that are related to the total dose are not the norm with PDT, also, little or no scarring occurs with a moderate healing process. Recent progress in ALA-PDT treatment revealed successful elimination of ATL leukemic cells with the highly-specific leukemia cell death via apoptosis and/or necrosis with minimal damage to the normal PBMCs even in whole blood specimens; indicating the possibility that ALA-PDT/PDD can inhibit the progression of ATL from indolent to aggressive types. Moreover, ATL intermediate pre-leukemic cells as well as leukemic cells were sensitively detected by ALA-PDD for precise diagnosis, suggesting the possibility of ALA-PDT/PDD use as preemptive therapy of ATL since it eliminates the small population of leukemic and/or pre-leukemic cells in high-risk indolent ATL before the overt onset of aggressive ATL. Additionally, there is an optimistic expectation for the elimination of leukemic and/or pre-leukemic ATL cell population from high-risk ACs with ALA-PDT/PDD. Along with the extracorporeal circulation system, ALA-PDT/PDD may facilitate the early diagnosis and treatment of various malignancies besides ATL, including other lymphoid/myeloid leukemia types. Furthermore, aberrantly-activated cells in non-malignant diseases such as chronic inflammatory diseases, auto-immune diseases, GVHDs, transplantation rejection could be targeted. Circulating tumor cells (CTCs) in the peripheral blood, which are responsible for tumor metastasis from primary cancers and sarcomas, could also be targeted with this treatment.

To optimize several PDT-related parameters such as photosensitizer and light doses, photosensitizer and light sources, drug-light intervals, fluence rates, in combination with chemotherapy in order to enhance the effectiveness of the tumor-killing activity, and also to enhance the innate and adaptive anti-tumor immune responses with optimization of immunogenic cell death, further research is required.
